# Supplementary material for: Bayesian refinement of protein structures and ensembles against SAXS data using molecular dynamics
Source: PLoS Comput Biol. 2017 Oct 18;13(10):e1005800. doi: 10.1371/journal.pcbi.1005800 (PMC5662244; doi:10.1371/journal.pcbi.1005800)
Supplement: S1 Archive — (BZ2) [file pcbi.1005800.s011.tar.bz2 › gromacs_4.62_bayesian_saxs_refi/share/html/online.html]

GROMACS 4.6 Online Reference 


| GROMACS 4.6  Online Reference |

**VERSION 4.6  
Sat 19 Jan 2013**

---

|  |  |
| --- | --- |
| General Getting Started    Flow Chart    File Formats    mdp options    FAQ | Programs  Options    do\_dssp  editconf  eneconv  g\_anadock  g\_anaeig  g\_analyze  g\_angle  g\_bar  g\_bond  g\_bundle  g\_chi  g\_cluster  g\_clustsize  g\_confrms  g\_covar  g\_current  g\_density  g\_densmap  g\_densorder  g\_dielectric  g\_dih  g\_dipoles  g\_disre  g\_dist  g\_dos  g\_dyecoupl  g\_dyndom  genbox  genconf  g\_enemat  g\_energy  genion  genrestr  g\_filter  g\_gyrate  g\_h2order  g\_hbond  g\_helix  g\_helixorient  g\_hydorder  g\_kinetics  g\_lie  g\_luck  g\_mdmat  g\_membed  g\_mindist  g\_morph  g\_msd  gmxcheck  gmxdump  g\_nmeig  g\_nmens  g\_nmtraj  g\_options  g\_order  g\_pme\_error  g\_polystat  g\_potential  g\_principal  g\_protonate  g\_rama  g\_rdf  g\_rms  g\_rmsdist  g\_rmsf  grompp  g\_rotacf  g\_rotmat  g\_saltbr  g\_sans  g\_sas  g\_select  g\_sgangle  g\_sham  g\_sigeps  g\_sorient  g\_spatial  g\_spol  g\_tcaf  g\_traj  g\_tune\_pme  g\_vanhove  g\_velacc  g\_wham  g\_wheel  g\_x2top  make\_edi  make\_ndx  mdrun  mk\_angndx  pdb2gmx  tpbconv  trjcat  trjconv  trjorder  xpm2ps |

---

### Programs by Topic

Generating topologies and coordinates  
Running a simulation  
Viewing trajectories  
Processing energies  
Converting files  
Tools  
Distances between structures  
Distances in structures over time  
Mass distribution properties over time  
Analyzing bonded interactions  
Structural properties  
Kinetic properties  
Electrostatic properties  
Protein-specific analysis  
Interfaces  
Covariance analysis  
Normal modes  


|  |  |  |  |  |  |  |  |  |  |  |  |  |  |  |  |  |  |  |
| --- | --- | --- | --- | --- | --- | --- | --- | --- | --- | --- | --- | --- | --- | --- | --- | --- | --- | --- |
| \|  \|  \|  \|  \|  \|  \|  \|  \|  \|  \|  \|  \|  \|  \|  \|  \|  \|  \| \| --- \| --- \| --- \| --- \| --- \| --- \| --- \| --- \| --- \| --- \| --- \| --- \| --- \| --- \| --- \| --- \| --- \| --- \| \| **Generating topologies and coordinates**\| editconf \| edits the box and writes subgroups \|\| g\_protonate \| protonates structures \|\| g\_x2top \| generates a primitive topology from coordinates \|\| genbox \| solvates a system \|\| genconf \| multiplies a conformation in 'random' orientations \|\| genion \| generates mono atomic ions on energetically favorable positions \|\| genrestr \| generates position restraints or distance restraints for index groups \|\| pdb2gmx \| converts coordinate files to topology and FF-compliant coordinate files \| \| \| |

|  |  |  |  |  |  |  |  |  |
| --- | --- | --- | --- | --- | --- | --- | --- | --- |
| \|  \|  \|  \|  \|  \|  \|  \|  \| \| --- \| --- \| --- \| --- \| --- \| --- \| --- \| --- \| \| **Running a simulation**\| grompp \| makes a run input file \|\| mdrun \| performs a simulation, do a normal mode analysis or an energy minimization \|\| tpbconv \| makes a run input file for restarting a crashed run \| \| \| |

|  |  |  |  |  |  |  |
| --- | --- | --- | --- | --- | --- | --- |
| \|  \|  \|  \|  \|  \|  \| \| --- \| --- \| --- \| --- \| --- \| --- \| \| **Viewing trajectories**\| g\_nmtraj \| generate a virtual trajectory from an eigenvector \|\| ngmx \| displays a trajectory \| \| \| |

|  |  |  |  |  |  |  |  |  |
| --- | --- | --- | --- | --- | --- | --- | --- | --- |
| \|  \|  \|  \|  \|  \|  \|  \|  \| \| --- \| --- \| --- \| --- \| --- \| --- \| --- \| --- \| \| **Processing energies**\| g\_enemat \| extracts an energy matrix from an energy file \|\| g\_energy \| writes energies to xvg files and displays averages \|\| mdrun \| with -rerun (re)calculates energies for trajectory frames \| \| \| |

|  |  |  |  |  |  |  |  |  |  |  |  |  |  |  |
| --- | --- | --- | --- | --- | --- | --- | --- | --- | --- | --- | --- | --- | --- | --- |
| \|  \|  \|  \|  \|  \|  \|  \|  \|  \|  \|  \|  \|  \|  \| \| --- \| --- \| --- \| --- \| --- \| --- \| --- \| --- \| --- \| --- \| --- \| --- \| --- \| --- \| \| **Converting files**\| editconf \| converts and manipulates structure files \|\| eneconv \| converts energy files \|\| g\_sigeps \| convert c6/12 or c6/cn combinations to and from sigma/epsilon \|\| trjcat \| concatenates trajectory files \|\| trjconv \| converts and manipulates trajectory files \|\| xpm2ps \| converts XPM matrices to encapsulated postscript (or XPM) \| \| \| |

|  |  |  |  |  |  |  |  |  |  |  |  |  |  |  |  |  |  |  |  |  |  |  |  |  |  |  |  |  |  |  |  |  |  |  |  |  |  |  |
| --- | --- | --- | --- | --- | --- | --- | --- | --- | --- | --- | --- | --- | --- | --- | --- | --- | --- | --- | --- | --- | --- | --- | --- | --- | --- | --- | --- | --- | --- | --- | --- | --- | --- | --- | --- | --- | --- | --- |
| \|  \|  \|  \|  \|  \|  \|  \|  \|  \|  \|  \|  \|  \|  \|  \|  \|  \|  \|  \|  \|  \|  \|  \|  \|  \|  \|  \|  \|  \|  \|  \|  \|  \|  \|  \|  \|  \|  \| \| --- \| --- \| --- \| --- \| --- \| --- \| --- \| --- \| --- \| --- \| --- \| --- \| --- \| --- \| --- \| --- \| --- \| --- \| --- \| --- \| --- \| --- \| --- \| --- \| --- \| --- \| --- \| --- \| --- \| --- \| --- \| --- \| --- \| --- \| --- \| --- \| --- \| --- \| \| **Tools**\| g\_analyze \| analyzes data sets \|\| g\_dyndom \| interpolate and extrapolate structure rotations \|\| g\_filter \| frequency filters trajectories, useful for making smooth movies \|\| g\_lie \| free energy estimate from linear combinations \|\| g\_morph \| linear interpolation of conformations \|\| g\_pme\_error \| estimates the error of using PME with a given input file \|\| g\_select \| selects groups of atoms based on flexible textual selections \|\| g\_sham \| read/write xmgr and xvgr data sets \|\| g\_spatial \| calculates the spatial distribution function \|\| g\_traj \| plots x, v and f of selected atoms/groups (and more) from a trajectory \|\| g\_tune\_pme \| time mdrun as a function of PME nodes to optimize settings \|\| g\_wham \| weighted histogram analysis after umbrella sampling \|\| gmxcheck \| checks and compares files \|\| gmxdump \| makes binary files human readable \|\| make\_ndx \| makes index files \|\| mk\_angndx \| generates index files for g\_angle \|\| trjorder \| orders molecules according to their distance to a group \|\| xpm2ps \| convert XPM (XPixelMap) file to postscript \| \| \| |

|  |  |  |  |  |  |  |  |  |  |  |
| --- | --- | --- | --- | --- | --- | --- | --- | --- | --- | --- |
| \|  \|  \|  \|  \|  \|  \|  \|  \|  \|  \| \| --- \| --- \| --- \| --- \| --- \| --- \| --- \| --- \| --- \| --- \| \| **Distances between structures**\| g\_cluster \| clusters structures \|\| g\_confrms \| fits two structures and calculates the rmsd \|\| g\_rms \| calculates rmsd's with a reference structure and rmsd matrices \|\| g\_rmsf \| calculates atomic fluctuations \| \| \| |

|  |  |  |  |  |  |  |  |  |  |  |  |  |  |  |
| --- | --- | --- | --- | --- | --- | --- | --- | --- | --- | --- | --- | --- | --- | --- |
| \|  \|  \|  \|  \|  \|  \|  \|  \|  \|  \|  \|  \|  \|  \| \| --- \| --- \| --- \| --- \| --- \| --- \| --- \| --- \| --- \| --- \| --- \| --- \| --- \| --- \| \| **Distances in structures over time**\| g\_bond \| calculates distances between atoms \|\| g\_dist \| calculates the distances between the centers of mass of two groups \|\| g\_mindist \| calculates the minimum distance between two groups \|\| g\_mdmat \| calculates residue contact maps \|\| g\_polystat \| calculates static properties of polymers \|\| g\_rmsdist \| calculates atom pair distances averaged with power -2, -3 or -6 \| \| \| |

|  |  |  |  |  |  |  |  |  |  |  |  |  |  |  |  |  |  |  |  |  |
| --- | --- | --- | --- | --- | --- | --- | --- | --- | --- | --- | --- | --- | --- | --- | --- | --- | --- | --- | --- | --- |
| \|  \|  \|  \|  \|  \|  \|  \|  \|  \|  \|  \|  \|  \|  \|  \|  \|  \|  \|  \|  \| \| --- \| --- \| --- \| --- \| --- \| --- \| --- \| --- \| --- \| --- \| --- \| --- \| --- \| --- \| --- \| --- \| --- \| --- \| --- \| --- \| \| **Mass distribution properties over time**\| g\_gyrate \| calculates the radius of gyration \|\| g\_msd \| calculates mean square displacements \|\| g\_polystat \| calculates static properties of polymers \|\| g\_rdf \| calculates radial distribution functions \|\| g\_rotacf \| calculates the rotational correlation function for molecules \|\| g\_rotmat \| plots the rotation matrix for fitting to a reference structure \|\| g\_sans \| computes the small angle neutron scattering spectra \|\| g\_traj \| plots x, v, f, box, temperature and rotational energy \|\| g\_vanhove \| calculates Van Hove displacement functions \| \| \| |

|  |  |  |  |  |  |  |  |  |
| --- | --- | --- | --- | --- | --- | --- | --- | --- |
| \|  \|  \|  \|  \|  \|  \|  \|  \| \| --- \| --- \| --- \| --- \| --- \| --- \| --- \| --- \| \| **Analyzing bonded interactions**\| g\_angle \| calculates distributions and correlations for angles and dihedrals \|\| g\_bond \| calculates bond length distributions \|\| mk\_angndx \| generates index files for g\_angle \| \| \| |

|  |  |  |  |  |  |  |  |  |  |  |  |  |  |  |  |  |  |  |  |  |  |  |  |  |  |  |  |  |
| --- | --- | --- | --- | --- | --- | --- | --- | --- | --- | --- | --- | --- | --- | --- | --- | --- | --- | --- | --- | --- | --- | --- | --- | --- | --- | --- | --- | --- |
| \|  \|  \|  \|  \|  \|  \|  \|  \|  \|  \|  \|  \|  \|  \|  \|  \|  \|  \|  \|  \|  \|  \|  \|  \|  \|  \|  \|  \| \| --- \| --- \| --- \| --- \| --- \| --- \| --- \| --- \| --- \| --- \| --- \| --- \| --- \| --- \| --- \| --- \| --- \| --- \| --- \| --- \| --- \| --- \| --- \| --- \| --- \| --- \| --- \| --- \| \| **Structural properties**\| g\_anadock \| cluster structures from Autodock runs \|\| g\_bundle \| analyzes bundles of axes, e.g. helices \|\| g\_clustsize \| calculate size distributions of atomic clusters \|\| g\_disre \| analyzes distance restraints \|\| g\_hbond \| computes and analyzes hydrogen bonds \|\| g\_order \| computes the order parameter per atom for carbon tails \|\| g\_principal \| calculates axes of inertia for a group of atoms \|\| g\_rdf \| calculates radial distribution functions \|\| g\_saltbr \| computes salt bridges \|\| g\_sas \| computes solvent accessible surface area \|\| g\_sgangle \| computes the angle and distance between two groups \|\| g\_sorient \| analyzes solvent orientation around solutes \|\| g\_spol \| analyzes solvent dipole orientation and polarization around solutes \| \| \| |

|  |  |  |  |  |  |  |  |  |  |  |  |  |  |  |  |  |  |  |  |  |  |  |
| --- | --- | --- | --- | --- | --- | --- | --- | --- | --- | --- | --- | --- | --- | --- | --- | --- | --- | --- | --- | --- | --- | --- |
| \|  \|  \|  \|  \|  \|  \|  \|  \|  \|  \|  \|  \|  \|  \|  \|  \|  \|  \|  \|  \|  \|  \| \| --- \| --- \| --- \| --- \| --- \| --- \| --- \| --- \| --- \| --- \| --- \| --- \| --- \| --- \| --- \| --- \| --- \| --- \| --- \| --- \| --- \| --- \| \| **Kinetic properties**\| g\_bar \| calculates free energy difference estimates through Bennett's acceptance ratio \|\| g\_current \| calculate current autocorrelation function of system \|\| g\_dos \| analyzes density of states and properties based on that \|\| g\_dyecoupl \| extracts dye dynamics from trajectories \|\| g\_kinetics \| analyzes kinetic constants from properties based on the Eyring model \|\| g\_principal \| calculate principal axes of inertion for a group of atoms \|\| g\_tcaf \| calculates viscosities of liquids \|\| g\_traj \| plots x, v, f, box, temperature and rotational energy \|\| g\_vanhove \| compute Van Hove correlation function \|\| g\_velacc \| calculates velocity autocorrelation functions \| \| \| |

|  |  |  |  |  |  |  |  |  |  |  |  |  |  |  |
| --- | --- | --- | --- | --- | --- | --- | --- | --- | --- | --- | --- | --- | --- | --- |
| \|  \|  \|  \|  \|  \|  \|  \|  \|  \|  \|  \|  \|  \|  \| \| --- \| --- \| --- \| --- \| --- \| --- \| --- \| --- \| --- \| --- \| --- \| --- \| --- \| --- \| \| **Electrostatic properties**\| g\_current \| calculates dielectric constants for charged systems \|\| g\_dielectric \| calculates frequency dependent dielectric constants \|\| g\_dipoles \| computes the total dipole plus fluctuations \|\| g\_potential \| calculates the electrostatic potential across the box \|\| g\_spol \| analyze dipoles around a solute \|\| genion \| generates mono atomic ions on energetically favorable positions \| \| \| |

|  |  |  |  |  |  |  |  |  |  |  |  |  |  |  |  |  |
| --- | --- | --- | --- | --- | --- | --- | --- | --- | --- | --- | --- | --- | --- | --- | --- | --- |
| \|  \|  \|  \|  \|  \|  \|  \|  \|  \|  \|  \|  \|  \|  \|  \|  \| \| --- \| --- \| --- \| --- \| --- \| --- \| --- \| --- \| --- \| --- \| --- \| --- \| --- \| --- \| --- \| --- \| \| **Protein-specific analysis**\| do\_dssp \| assigns secondary structure and calculates solvent accessible surface area \|\| g\_chi \| calculates everything you want to know about chi and other dihedrals \|\| g\_helix \| calculates basic properties of alpha helices \|\| g\_helixorient \| calculates local pitch/bending/rotation/orientation inside helices \|\| g\_rama \| computes Ramachandran plots \|\| g\_wheel \| plots helical wheels \|\| g\_xrama \| shows animated Ramachandran plots \| \| \| |

|  |  |  |  |  |  |  |  |  |  |  |  |  |  |  |  |  |  |  |  |  |
| --- | --- | --- | --- | --- | --- | --- | --- | --- | --- | --- | --- | --- | --- | --- | --- | --- | --- | --- | --- | --- |
| \|  \|  \|  \|  \|  \|  \|  \|  \|  \|  \|  \|  \|  \|  \|  \|  \|  \|  \|  \|  \| \| --- \| --- \| --- \| --- \| --- \| --- \| --- \| --- \| --- \| --- \| --- \| --- \| --- \| --- \| --- \| --- \| --- \| --- \| --- \| --- \| \| **Interfaces**\| g\_bundle \| analyzes bundles of axes, e.g. transmembrane helices \|\| g\_density \| calculates the density of the system \|\| g\_densmap \| calculates 2D planar or axial-radial density maps \|\| g\_densorder \| calculate surface fluctuations \|\| g\_h2order \| computes the orientation of water molecules \|\| g\_hydorder \| computes tetrahedrality parameters around a given atom \|\| g\_order \| computes the order parameter per atom for carbon tails \|\| g\_membed \| embeds a protein into a lipid bilayer \|\| g\_potential \| calculates the electrostatic potential across the box \| \| \| |

|  |  |  |  |  |  |  |  |  |
| --- | --- | --- | --- | --- | --- | --- | --- | --- |
| \|  \|  \|  \|  \|  \|  \|  \|  \| \| --- \| --- \| --- \| --- \| --- \| --- \| --- \| --- \| \| **Covariance analysis**\| g\_anaeig \| analyzes the eigenvectors \|\| g\_covar \| calculates and diagonalizes the covariance matrix \|\| make\_edi \| generate input files for essential dynamics sampling \| \| \| |

|  |  |  |  |  |  |  |  |  |  |  |  |  |  |  |
| --- | --- | --- | --- | --- | --- | --- | --- | --- | --- | --- | --- | --- | --- | --- |
| \|  \|  \|  \|  \|  \|  \|  \|  \|  \|  \|  \|  \|  \|  \| \| --- \| --- \| --- \| --- \| --- \| --- \| --- \| --- \| --- \| --- \| --- \| --- \| --- \| --- \| \| **Normal modes**\| g\_anaeig \| analyzes the normal modes \|\| g\_nmeig \| diagonalizes the Hessian \|\| g\_nmtraj \| generate oscillating trajectory of an eigenmode \|\| g\_nmens \| generates an ensemble of structures from the normal modes \|\| grompp \| makes a run input file \|\| mdrun \| finds a potential energy minimum and calculates the Hessian \| \| \| |

---

http://www.gromacs.org
